# Screening of risk factors and construction of a prediction model for enteral nutrition intolerance in elderly patients with comorbidities and sarcopenia

**DOI:** 10.3389/fnut.2026.1917057

**Published:** 2026-10-01

**Authors:** Hao Wang, Ruichun Lu

**Affiliations:** 1 Department of Radiation Oncology, Qingdao Municipal Hospital, Qingdao, China; 2 Department of Geriatrics, Qingdao Municipal Hospital, Qingdao, China

**Keywords:** comorbidity, enteral nutrition, nomogram, risk factors, sarcopenia

## Abstract

**Objective:**

To screen the independent risk factors for enteral nutrition intolerance (ENI) in elderly comorbid patients with sarcopenia, and to establish and validate a clinical prediction model for individualized risk assessment and targeted intervention.

**Methods:**

A total of 202 elderly comorbid patients with sarcopenia receiving enteral nutrition were retrospectively enrolled from the Geriatrics Department of Qingdao Municipal Hospital between January 2024 and December 2025. Patients were divided into ENI group (*n* = 108) and tolerant group (*n* = 94). Potential predictive indicators were initially screened via univariate logistic regression. Statistically significant variables were then included in multivariable logistic regression to determine independent risk factors for target events. A visual nomogram prediction model was developed incorporating these independent predictors. Four statistical methods were applied to fully assess model performance: ROC curves for discrimination evaluation, calibration curves for risk fitting verification, DCA for clinical utility assessment, and SHAP analysis to interpret the predictive weight of each variable.

**Results:**

Severe dependence in activities of daily living (ADL) and concomitant psychiatric disorders were independently linked to higher ENI risk, whereas red blood cell (RBC) levels produced a protective effect. The predictive model delivered solid discriminative performance, registering an AUC of 0.77 (95% CI: 0.69–0.85) in the training cohort and 0.70 (95% CI: 0.57–0.84) in the validation cohort. The Hosmer–Lemeshow goodness-of-fit test confirmed adequate model calibration (*p* > 0.05), and net clinical benefit analysis validated steady clinical value across clinically relevant risk threshold ranges. SHAP analysis indicated that concomitant psychiatric disorders served as the primary variable driving the model’s predictive results.

**Conclusion:**

The developed nomogram exhibited acceptable discrimination and acceptable calibration upon internal validation, with detectable net clinical benefit. This visual risk scoring tool enables personalized ENI risk estimation in elderly multimorbid patients with sarcopenia. Additional multicenter external validation work should be finished prior to clinical adoption.

## Introduction

Concurrent chronic diseases and sarcopenia represent critical health burdens for elderly hospitalized populations. The prevalence of sarcopenia is markedly higher among the oldest-old inpatients (1). As outlined in the 2025 consensus statement from the Asian Working Group for Sarcopenia (AWGS), sarcopenia is a progressive, age-associated muscle disorder characterized by gradual loss of skeletal muscle mass and deterioration of muscular strength (2). This condition diminishes physical function in older adults and markedly increases their risks of physical disability, repeated hospital readmission, and long-term all-cause mortality (2). Cumulative population-based studies confirm that sarcopenia brings severe public health burdens to the elderly, and its prevalence differs significantly across age strata and diverse care settings. Pooled statistics derived from recent systematic reviews indicate sarcopenia affects 16.5% of community-residing Asian individuals aged 60 years and older (3), whereas the figure rises markedly to 26.3%–65.9% among older patients residing in hospitals and nursing facilities (4). From a clinical perspective, sarcopenia coexisting with multiple chronic illnesses and undernutrition creates a mutually reinforcing vicious loop among ageing patients. The two pathological states exacerbate each other continuously, gradually weakening physical performance and ultimately worsening long-term prognostic outcomes for older inpatients (5).

Current ESPEN clinical guidelines consistently recommend enteral nutrition (EN) as the primary nutritional approach for older multimorbid patients with sarcopenia (6–8). For patients retaining intact digestive tract function, enteral nutrition can substantially improve overall nutritional conditions, maintain skeletal muscle volume, and slow the progressive worsening of malnutrition alongside physical frailty (9). That said, gastrointestinal disorders, compromised intestinal mucosal integrity and additional contributing conditions can trigger enteral nutrition intolerance (ENI), with its prevalence ranging between 40% and 60% (10). Typical clinical manifestations include diarrhoea, abdominal bloating and other related gastrointestinal complaints (11). This adverse clinical event worsens sarcopenia and undernutrition, while elevating hazards of infectious complications, all-cause death and excess medical expenditure.

Available clinical research rarely delivers targeted analyses of ENI in elderly patients with comorbidities combined with sarcopenia. Few studies have systematically screened and quantified independent risk factors unique to this complex geriatric population. Most predictive tools for assessing ENI are constructed for critically ill patients or patients with malignant tumors. Such prediction tools cannot be directly applied to sarcopenic elderly patients with multiple chronic diseases (12–15). These limitations hinder early risk identification and individualized nutritional management in clinical practice, resulting in distinct research gaps and a shortage of specialized assessment tools for this vulnerable patient group.

The current research was designed to filter factors independently linked to ENI occurrence among the target group. We further developed and rigorously validated a risk prediction tool based on these predictors. The derived findings can guide clinicians to carry out layered clinical interventions and tailor exclusive enteral nutrition schemes, with the goal of boosting overall patient clinical benefits (16).

## Subjects and methods

### Study subjects

Two hundred two elderly patients diagnosed with concurrent multimorbidity and sarcopenia who accepted EN intervention in the Geriatric Ward of Qingdao Municipal Hospital during the period spanning January 2024 to December 2025 were recruited for this study. All recruited participants were subjected to stringent screening procedures in compliance with formal standardized enrollment, elimination and trial termination criteria. Ethical clearance for this observational retrospective cohort study was granted by the Ethics Committee of Qingdao Municipal Hospital (certificate No. 2025-KY-047).

Every enrolled subject underwent rigorous eligibility assessment following prespecified standards for entry, exclusion and study discontinuation. Entry standards: 1. Age ≥60 years; 2. Two or more concurrent chronic comorbidities (hypertension, diabetes, coronary heart disease, chronic obstructive pulmonary disease, etc.); 3. Sarcopenia confirmed through objective testing. Skeletal muscle mass was measured using DXA or BIA, and handgrip strength was evaluated with a hand dynamometer; 4. The anticipated enteral nutrition course lasted no less than 72 h, which allowed full observation of symptoms related to feeding intolerance; 5. Comprehensive clinical medical records of subjects were fully accessible; 6. Every participant joined the present study voluntarily. Prior to formal recruitment, written informed consent forms signed by each subject or their legal authorized representative were obtained. All enrolled patients received EN strictly following ASPEN clinical practice guidelines (17). All subjects had intact gastrointestinal tracts, the core prerequisite for enteral feeding. The specific EN initiation indications in our center were categorized as follows (18): The specific EN initiation indications in our center were categorized as follows (18): 1. Dysphagia with impaired oral feeding capacity; 2. Insufficient oral intake secondary to central nervous system damage; 3. Severe malnutrition confirmed by nutritional assessment; 4. High nutritional risk identified via validated screening tools; 5. Frailty and physical dysfunction preventing adequate spontaneous oral intake. Exclusion criteria: 1. Severe gastrointestinal disorders such as gastrointestinal obstruction or active bleeding; 2. Severe underlying diseases including severe hepatic or renal failure and end-stage malignant tumor; 3. History of abdominal surgery within the previous 3 months; 4. Inability to cooperate with the relevant assessments and data collection procedures.

### Clinical data

Basic demographic indicators including age, sex and BMI were extracted from medical charts. All medication records were sorted and archived separately. Concomitant psychiatric disorders referred to cognitive or mood disorders confirmed by clinical diagnosis according to hospital admission files. Cognitive conditions covered Alzheimer’s disease and vascular dementia, while mood conditions mainly included depressive and anxious disorders. Activities of daily living (ADL) score and Charlson Comorbidity Index (CCI) were also evaluated.

Venous blood samples were collected after overnight fasting to detect routine laboratory parameters, including white blood cell count, red blood cell count, C-reactive protein (CRP), hemoglobin (Hb), albumin, blood glucose, and vitamin D. All enteral feeding parameters and clinical factors related to enteral nutrition tolerance were systematically collected from complete electronic medical records, including enteral formula type, feeding volume, feeding initiation time, nutritional access route, active infection status, antibiotic use, and administration of acid-suppressive agents, and prokinetic drugs. All regimen adjustments during enteral nutrition treatment were fully documented. All clinical data in this observational retrospective cohort study were extracted from electronic medical records. The missing proportion of each predictor and outcome variable was calculated. Complete-case analysis was applied in the final statistical analysis. Participants with missing values for any included variable were excluded from model construction, and no multiple imputation was performed in this study.

### Sarcopenia and functional parameters

All participants underwent standardized sarcopenia screening and diagnostic evaluation based on the 2019 consensus guidelines established by AWGS (19). Calf circumference (CC) was adopted as the preliminary screening indicator to identify potential sarcopenia (20). Participants who screened positive (CC < 34 cm for men and <33 cm for women) underwent further examinations covering muscle strength, physical performance, and appendicular skeletal muscle mass to confirm the final diagnosis. Dominant-hand grip strength was measured to evaluate muscular strength, with gender-specific threshold values of <28.0 kg in males and <18.0 kg in females. Physical functional impairment was determined by two routine physical tests: a usual 6-meter walking speed slower than 1.0 m/s or a 5-repetition sit-to-stand test requiring no less than 12 s to complete. Appendicular skeletal muscle mass index (ASMI) was measured using dual-energy X-ray absorptiometry (DXA) and bioelectrical impedance analysis (BIA), with exclusive cutoffs applicable to Asian populations. For DXA measurement, the low muscle mass thresholds were <7.0 kg/m^2^ for males and <5.4 kg/m^2^ for females; for BIA measurement, the corresponding cutoffs were <7.0 kg/m^2^ and <5.7 kg/m^2^ for males and females, respectively. The definitive diagnosis of sarcopenia was made when reduced skeletal muscle mass was accompanied by diminished muscle strength or impaired physical ability. Participants with simultaneous deficits in muscle mass, muscle strength, and physical function were categorized into the severe sarcopenia group.

### Assessment of ENI

We set uniform diagnostic benchmarks for ENI identification. The evaluation indicators for ENI in this study were referenced to the 2022 clinical practice guidelines for nutritional support in critically ill patients issued by the American Society for Parenteral and Enteral Nutrition (ASPEN) (21). All digestive adverse events emerging during enteral nutrition support, such as nausea, emesis, liquid stool, abdominal bloating and epigastric discomfort, were fully documented and stratified into three tiers (mild, moderate and severe) according to clinical presentations. The lasting time of every recorded symptom was retrieved retrospectively from electronic medical charts, with duration converted to hours or days for quantitative analysis. We gathered core clinical information related to enteral nutrition interventions, such as temporary interruption of enteral feeding, cutbacks in feeding dosage, and prescription of gastrointestinal prokinetics to alleviate digestive tract disturbances. Gastric residual volume (GRV) was routinely monitored every 6 hours, with pathological gastric retention defined as GRV ≥ 500 mL within 6 hours; the occurrence of aspiration events was systematically documented for all enrolled patients. To avoid inaccurate judgment of enteral nutrition intolerance, every case of liquid stool was subjected to detailed differential screening. Researchers reviewed lab test indicators, patients’ medication records and clinical diagnostic conclusions to separate infectious, drug-triggered and formula-associated diarrheal conditions. Within the present research, feeding intolerance related to enteral nutrition was identified by the following criteria: moderate or severe digestive complaints, pathological gastric residual volume exceeding 500 mL within 6 hours, or aspiration incidents demanding clinical intervention. Based on the above standardized assessment framework, elderly multimorbid patients with sarcopenia receiving EN were stratified into the EN tolerance group and ENI group.

### Statistical methods

Statistical processing of all collected data was completed using R version 4.3.1. All statistical tests adopted two-tailed analysis, and any *p* value less than 0.05 was regarded as the cutoff for statistically meaningful differences. Baseline demographic and clinical indicators were categorized and reported in line with their distribution patterns. Normally distributed continuous metrics were expressed as mean ± standard deviation. Continuous variables with non-normal distribution were described by median and interquartile range. Count-based categorical clinical factors were summarized with absolute case numbers and corresponding proportions. Appropriate statistical tests including Mann–Whitney *U* test, Kruskal–Wallis *H* test, and *χ^2^* test were adopted for pairwise and multi-group inter-cohort comparisons. To screen effective predictive indicators for ENI, preliminary univariate logistic regression analysis was performed across all candidate covariates, and variables with a univariate *p*-value less than 0.10 were retained for subsequent analytical procedures. A hierarchical penalized least absolute shrinkage and selection operator (LASSO) logistic regression coupled with 10-fold cross-validation was adopted to reduce covariate dimensionality. Differentiated penalty weights were assigned based on the clinical significance of indicators: core clinical markers were set with a penalty factor of 0, while all remaining covariates received a penalty factor of 1. The optimal *λ.min* was identified by minimizing cross-validated binomial deviance, and covariates carrying non-zero regression coefficients under this *λ.min* were extracted as preliminarily screened predictors. All variables filtered via LASSO were further imported into a backward stepwise multivariate logistic regression model constructed based on the Akaike Information Criterion (AIC). Iterative elimination of covariates was conducted to minimize the AIC value and identify stable independent predictive factors of the study endpoint. The screening outcomes yielded by the two variable selection strategies (univariate analysis and LASSO penalized regression) were compared in the Results section to validate the robustness of covariate screening. The enrolled study population was randomly split into training and validation subgroups at a 7:3 ratio, supporting the subsequent construction of a visual ENI predictive nomogram based on the optimal regression model and internal verification of model performance. We assessed the newly constructed prediction model from four core evaluation perspectives. Receiver operating characteristic curve analysis together with the corresponding AUC values was adopted to measure how well this model could distinguish patients prone to enteral nutrition intolerance. Calibration curves and the Hosmer–Lemeshow test were used to examine the consistency between model-predicted risks and real clinical endpoints. To reduce overfitting bias and stabilize the model’s predictive robustness, we performed bootstrap resampling repeated 1,000 times. Decision curve analysis (DCA) was carried out to calculate the net clinical benefit at different risk thresholds, so as to validate the model’s practical clinical application value. Furthermore, SHapley Additive exPlanations *(SHAP)* analysis was utilized to quantitatively distinguish the independent predictive contribution and weight of each clinical variable included in the model.

## Results

### Comparison of baseline characteristics of study participants

A total of 202 elderly patients with comorbidities and sarcopenia were enrolled and assigned to the ENI group (*n* = 108) and the tolerance group (*n* = 94) based on the occurrence of ENI. Between-group comparison showed that age, BMI, RBC, CRP, albumin, ADL, and concomitant psychiatric disorders differed significantly (*p* < 0.05). The ENI group was older and had higher CRP levels, while BMI, RBC and albumin levels were lower than those in the tolerance group. Moreover, patients in the ENI group presented more severe ADL impairment and a higher proportion of concomitant psychiatric disorders. No significant intergroup differences were observed in WBC, Hb, blood glucose, vitamin D, gender, CCI grade, medication history, enteral formula type, active infection status, antibiotic administration, use of acid-suppressive agents and prokinetic drugs, and nutritional access routes (all *p* > 0.05) (Table 1).

**Table 1:** Comparison of baseline characteristics of study participants.

Variables	Tolerance group (*n* = 94)	ENI group (*n* = 108)	Statistic	*p*
Age, years, M (Q₁, Q₃)	75.46 ± 8.22	80.47 ± 10.13	Z = −3.88	<0.001*
BMI, kg/m^2^, M (Q₁, Q₃)	25.56 (22.22, 27.66)	22.34 (19.40, 26.17)	Z = −3.17	0.002*
WBC, ×10^9^/L, M (Q₁, Q₃)	5.80 (4.87, 7.43)	6.17 (4.87, 7.72)	Z = −0.86	0.388
RBC, ×10^12^/L, M (Q₁, Q₃)	4.10 (3.29, 4.36)	3.49 (3.01, 4.17)	Z = −3.21	0.001*
Hb, g/L, M (Q₁, Q₃)	123.50 (105.00, 135.00)	118.50 (98.00, 133.00)	Z = −1.55	0.122
CRP, mg/L, M (Q₁, Q₃)	0.88 (0.50, 2.97)	2.34 (0.92, 8.13)	Z = −4.31	<0.001*
Albumin, g/L, M (Q₁, Q₃)	37.10 (32.66, 39.08)	34.26 (31.45, 37.88)	Z = −2.02	0.043*
Blood Glucose, mmol/L, M (Q₁, Q₃)	5.84 (4.85, 6.74)	5.85 (4.85, 7.05)	Z = −0.52	0.601
Vitamin D, ng/mL, M (Q₁, Q₃)	19.00 (16.00, 32.00)	20.00 (16.75, 36.00)	Z = −0.64	0.523
Gender, *n*(%)			χ^2^ = 0.43	0.511
Female	47 (50.00)	49 (45.37)		
Male	47 (50.00)	59 (54.63)		
CCI Grade, *n* (%)			χ^2^ = 0.33	0.565
Mild comorbidity (CCI = 1–2)	12 (12.77)	11 (10.19)		
Moderate comorbidity (CCI = 3–4)	82 (87.23)	97 (89.81)		
ADL, *n* (%)			χ^2^ = 25.62	<0.001*
Independent	69 (73.40)	41 (37.96)		
Partial dependence	11 (11.70)	26 (24.07)		
Full dependence	14 (14.89)	41 (37.96)		
Concomitant psychiatric disorders, *n* (%)			χ^2^ = 13.68	<0.001*
Absent	91 (96.81)	86 (79.63)		
Present	3 (3.19)	22 (20.37)		
Medicine history, *n* (%)			—	0.785
No antihypertensive, lipid-lowering, or hypoglycemic drugs	3 (3.19)	5 (4.63)		
Single drug use	24 (25.53)	22 (20.37)		
Combined use of two drugs	57 (60.64)	67 (62.04)		
Combined use of three drugs	10 (10.64)	14 (12.96)		
Enteral formula type, n (%)			χ^2^ = 1.17	0.761
Oral self-diet	71 (75.53)	81 (75.00)		
Short-peptide enteral nutrition	6 (6.38)	9 (8.33)		
Whole-protein enteral nutrition	12 (12.77)	10 (9.26)		
Combined short-peptide and whole-protein enteral nutrition	5 (5.32)	8 (7.41)		
Active infection status			χ^2^ = 0.06	0.809
No	35 (37.23)	42 (38.89)		
Yes	59 (62.77)	66 (61.11)		
Antibiotic administration			χ^2^ = 0.04	0.835
No	64 (68.09)	75 (69.44)		
Yes	30 (31.91)	33 (30.56)		
Acid-suppressive agents			χ^2^ = 0.28	0.598
No	88 (93.62)	99 (91.67)		
Yes	6 (6.38)	9 (8.33)		
Prokinetic drugs			χ^2^ = 1.92	0.166
No	37 (39.36)	53 (49.07)		
Yes	57 (60.64)	55 (50.93)		
Nutritional access routes			χ^2^ = 0.33	
Nasogastric tube	77 (81.91)	85 (78.70)		
Nasoenteric tube	17 (18.09)	23 (21.30)		

*p < 0.05 was considered statistically significant. Z: Mann–Whitney test, χ^2^: Chi-square test, −: Fisher exact M: Median, Q₁: 1st Quartile, Q₃: 3rd Quartile. Charlson Comorbidity Index (CCI) standard grading: 0 = no comorbidity, 1–2 = mild comorbidity, 3–4 = moderate comorbidity, ≥5 = severe comorbidity. No participants had CCI scores of 1 or 4 in this study. BMI, body mass index; WBC, white blood cell count; Hb, hemoglobin; CRP, C-reactive protein; RBC, red blood cell count; ADL, activities of daily living; CPD, concomitant psychiatric disorders; CCI, Charlson Comorbidity Index; ENI, enteral nutrition intolerance.

### Univariate logistic regression analysis of influencing factors for ENI in elderly comorbid patients with sarcopenia

All clinical and laboratory indicators related to the study were included in the univariate logistic regression analysis. The results indicated that age, ADL, concomitant psychiatric disorders, RBC and CRP were significantly correlated with the occurrence of ENI in elderly comorbid patients with sarcopenia (*p* < 0.05).

Advanced age acted as a risk factor for ENI (OR = 1.05, 95% CI: 1.01–1.09, *p* = 0.014). Taking full independence in ADL as the reference group, patients with partial dependence and full dependence exhibited a remarkably elevated risk of developing ENI (OR = 3.26, 95% CI: 1.33–8.00, *p* = 0.010; OR = 4.30, 95% CI: 1.85–9.99, *p* < 0.001). Patients with concomitant psychiatric disorders also had a significantly higher risk of ENI (OR = 9.78, 95% CI: 2.17–43.97, *p* = 0.003). RBC served as a protective factor against ENI (OR = 0.45, 95% CI: 0.27–0.76, *p* = 0.003). Elevated CRP level increased the risk of ENI (OR = 1.08, 95% CI: 1.01–1.15, *p* = 0.023).

By contrast, gender, CCI grade, medication history, active infection status, antibiotic administration, use of acid-suppressive agents and prokinetic drugs, nutritional access routes, enteral formula types, BMI, WBC, Hb, albumin, blood glucose and vitamin D showed no significant association with ENI in elderly comorbid patients with sarcopenia (all *p* > 0.05) (Table 2).

**Table 2:** Univariate logistic regression analysis of influencing factors for ENI in elderly comorbid patients with sarcopenia.

Variables	β	S.E	Z	*p*	OR (95% CI)
Gender					
Female					1.00 (Reference)
Male	0.06	0.34	0.17	0.866	1.06 (0.54 ~ 2.06)
CCI Grade					
Mild comorbidity (CCI = 1–2)					1.00 (Reference)
Moderate comorbidity (CCI = 3–4)	0.18	0.53	0.33	0.740	1.19 (0.42 ~ 3.38)
Concomitant psychiatric disorders					
Absent					1.00 (Reference)
Present	2.28	0.77	2.97	0.003*	9.78 (2.17 ~ 43.97)
Medicine history					
No antihypertensive, lipid-lowering, or hypoglycemic drugs					1.00 (Reference)
Single drug use	−0.57	0.81	−0.71	0.480	0.56 (0.12 ~ 2.76)
Combined use of two drugs	−0.32	0.76	−0.42	0.675	0.73 (0.16 ~ 3.24)
Combined use of three drugs	−0.26	0.89	−0.29	0.770	0.77 (0.14 ~ 4.39)
Active infection status					
No					1.00 (Reference)
Yes	0.03	0.34	0.09	0.928	1.03 (0.53 ~ 2.02)
Antibiotic administration					
No					1.00 (Reference)
Yes	0.12	0.37	0.34	0.738	1.13 (0.55 ~ 2.35)
Acid-suppressive agents					
No					1.00 (Reference)
Yes	0.38	0.75	0.50	0.617	1.46 (0.33 ~ 6.34)
Prokinetic drugs					
No					1.00 (Reference)
Yes	−0.48	0.34	−1.40	0.161	0.62 (0.31 ~ 1.21)
Nutritional access routes					
Nasogastric tube					1.00 (Reference)
Nasoenteric tube	0.29	0.41	0.71	0.481	1.33 (0.60 ~ 2.97)
Enteral formula types					
Oral self-diet					1.00 (Reference)
Short-peptide enteral nutrition	0.60	0.73	0.82	0.413	1.82 (0.43 ~ 7.66)
Whole-protein enteral nutrition	−0.34	0.54	−0.64	0.523	0.71 (0.25 ~ 2.04)
Combined short-peptide and whole-protein enteral nutrition	1.16	0.82	1.41	0.160	3.19 (0.63 ~ 16.05)
ADL					
Independent					1.00 (Reference)
Partial dependence	1.18	0.46	2.58	0.010*	3.26 (1.33 ~ 8.00)
Full dependence	1.46	0.43	3.39	<0.001*	4.30 (1.85 ~ 9.99)
Age	0.05	0.02	2.45	0.014*	1.05 (1.01 ~ 1.09)
BMI	−0.01	0.01	−1.58	0.114	0.99 (0.97 ~ 1.00)
WBC	0.18	0.09	1.93	0.054	1.20 (1.00 ~ 1.44)
RBC	−0.79	0.27	−2.97	0.003*	0.45 (0.27 ~ 0.76)
Hb	−0.01	0.01	−1.61	0.108	0.99 (0.97 ~ 1.00)
CRP	0.07	0.03	2.28	0.023*	1.08 (1.01 ~ 1.15)
Albumin	−0.06	0.04	−1.43	0.153	0.94 (0.86 ~ 1.02)
Blood Glucose	0.09	0.12	0.77	0.444	1.09 (0.87 ~ 1.37)
Vitamin D	−0.00	0.01	−0.40	0.686	1.00 (0.98 ~ 1.01)

*p < 0.05 was considered statistically significant. OR: Odds Ratio, CI: Confidence Interval. Charlson Comorbidity Index (CCI) standard grading: 0 = no comorbidity, 1–2 = mild comorbidity, 3–4 = moderate comorbidity, ≥5 = severe comorbidity. No participants had CCI scores of 1 or 4 in this study.

### Dimensionality reduction via hierarchical penalized LASSO regression

All covariates with *p* < 0.10 in univariate logistic regression were enrolled into hierarchical penalized LASSO logistic regression with 10-fold cross-validation for dimensionality reduction. Based on clinical significance, core clinical indicators were assigned a penalty factor of 0 to avoid complete coefficient shrinkage, while other covariates received a penalty factor of 1. The optimal regularization parameter *λ*min was determined by minimizing cross-validated binomial deviance (Figure 1). At *λ*min, six covariates retained non-zero LASSO regression coefficients, including all five core clinical predictors that were subsequently identified as independent risk factors in the AIC-based backward stepwise multivariate logistic regression model. BMI was additionally retained with marginal predictive effects and excluded from the final multivariate model, as it barely improved the overall model fitting. Detailed LASSO regression coefficients of the six screened variables were summarized in Supplementary Table S1. Consistent retention of the five core predictors across two variable selection strategies confirmed the robustness of key predictive factors for ENI.

**Figure 1:**
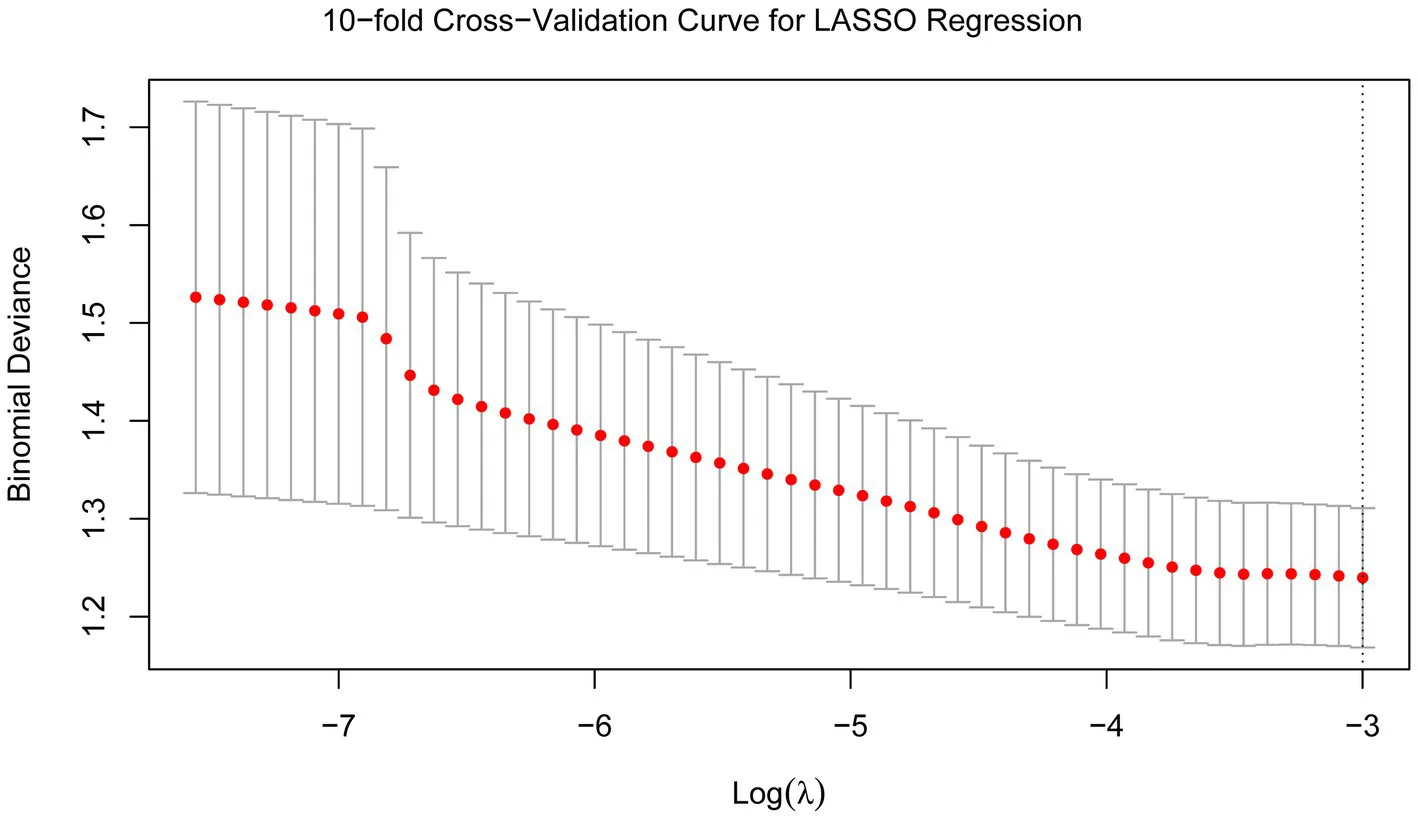
10-fold cross-validation curve for LASSO regression. The horizontal axis represents log-transformed penalty parameter log(*λ*), and the vertical axis denotes binomial deviance. The red dots indicate the mean cross-validated binomial deviance at corresponding log(*λ*) values, with vertical error bars showing standard error. The vertical dotted line marks the optimal *λ* value determined by 10-fold cross-validation for variable screening.

### Multivariate logistic regression analysis of independent influencing factors for ENI in elderly comorbid patients with sarcopenia

After preliminary screening via univariate logistic regression and subsequent dimension reduction using hierarchical penalized LASSO regression, the retained candidate variables were included in an AIC-based backward stepwise multivariate logistic regression model. The results showed that full ADL dependence, concomitant psychiatric disorders and RBC were independent influencing factors for ENI in elderly comorbid patients with sarcopenia (all *p* < 0.05). With full ADL independence as the reference, full ADL dependence was independently associated with a higher risk of ENI (OR = 2.97, 95% CI: 1.20–7.35, *p* = 0.018). The presence of concomitant psychiatric disorders also independently increased the risk of ENI (OR = 7.63, 95% CI: 1.54–37.75, *p* = 0.013). RBC emerged as an independent protective factor; each one-unit increase in RBC was related to a reduced likelihood of developing ENI (OR = 0.56, 95% CI: 0.32–0.99, *p* = 0.046). By comparison, advanced age, and elevated CRP levels were not independent predictors of ENI (all *p* > 0.05) (Table 3).

**Table 3:** Multivariate logistic regression analysis of independent influencing factors for ENI in elderly comorbid patients with sarcopenia.

Variables	β	S.E	Z	*p*	OR (95% CI)
Intercept	−0.61	2.37	−0.26	0.796	0.54 (0.01 ~ 56.91)
Concomitant psychiatric disorders					
Absent					1.00 (Reference)
Present	2.03	0.82	2.49	0.013*	7.63 (1.54 ~ 37.75)
ADL					
Independent					1.00 (Reference)
Partial dependence	0.55	0.52	1.07	0.285	1.74 (0.63 ~ 4.79)
Full dependence	1.09	0.46	2.36	0.018*	2.97 (1.20 ~ 7.35)
Age	0.03	0.03	0.86	0.388	1.03 (0.97 ~ 1.09)
RBC	−0.58	0.29	−2.00	0.046*	0.56 (0.32 ~ 0.99)
CRP	0.04	0.03	1.45	0.146	1.04 (0.99 ~ 1.11)

*p < 0.05 was considered statistically significant. OR, odds ratio; CI, confidence interval.

### Construction and performance evaluation of the prediction model for ENI in elderly comorbid patients with sarcopenia

#### Development of the nomogram for predicting ENI in elderly comorbid patients with sarcopenia

Based on the independent predictors obtained from multivariate logistic regression, we constructed a nomogram to quantify the risk of ENI in sarcopenic elderly multimorbid patients. Variable selection comprehensively considered clinical relevance, AIC and model performance (Figure 2). Univariate analysis revealed that age, RBC, CRP, ADL impairment, and concomitant psychiatric disorders were significantly correlated with ENI (all *p* < 0.05). Multivariate logistic regression further confirmed severe ADL dependence, concomitant psychiatric disorders, and decreased RBC as independent predictors of ENI (all *p* < 0.05). After balancing AIC optimization and clinical practicability, five variables were ultimately included in the nomogram: age, CRP, RBC, ADL impairment, and concomitant psychiatric disorders. Although age and CRP failed to emerge as independent predictors in multivariate regression, they were retained due to their ready clinical availability; their inclusion improved model fit and predictive stability. The corresponding logistic regression equation was as follows: logit (*P*) = −0.61 + 1.09 × severe ADL dependence + 2.03 × concomitant psychiatric disorders +0.03 × age−0.58 × RBC + 0.04 × CRP.

**Figure 2:**
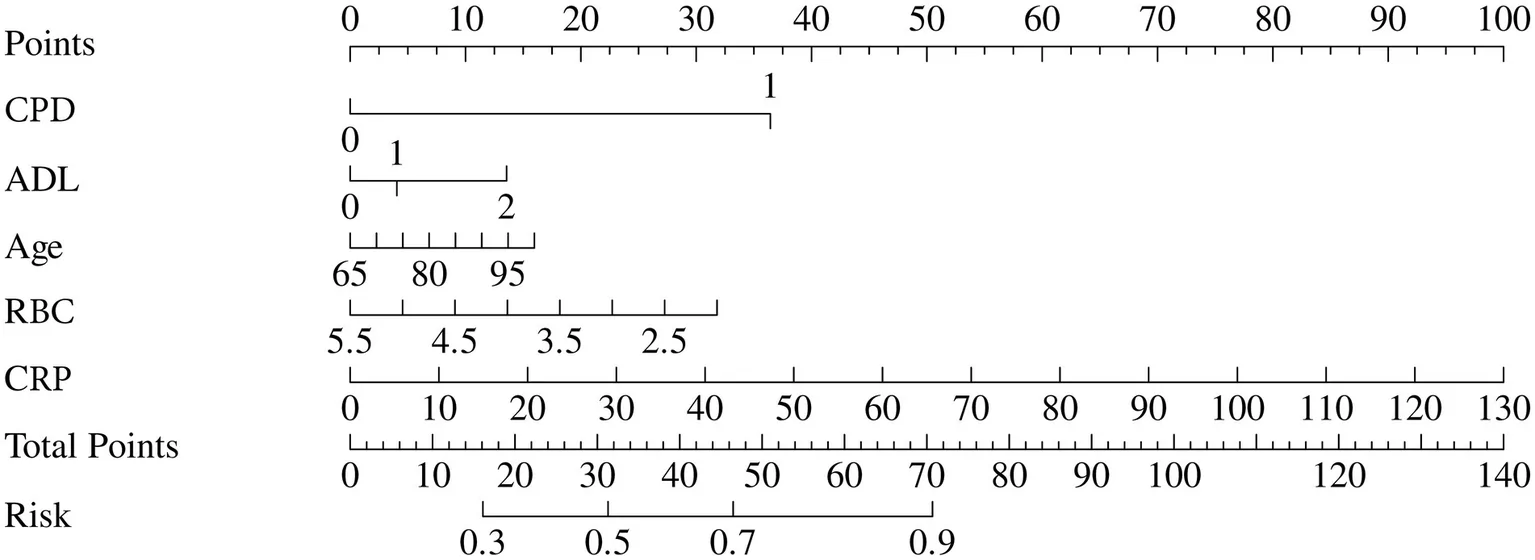
Nomogram for predicting ENI in elderly comorbid patients with sarcopenia. For each variable (ADL, CPD, age, RBC, and CRP), draw a vertical line upward from the patient’s measured value to the Points axis to obtain the corresponding score. The scores of all variables are summed to calculate the total points, and the predicted probability of ENI can be determined by projecting the total points to the Total Points Risk axis. ADL, activities of daily living; CPD, concomitant psychiatric disorders; CRP, C-reactive protein; ENI, enteral nutrition intolerance; RBC, red blood cell count.

#### Calibration ability

Model calibration was assessed using the Hosmer–Lemeshow (H-L) goodness-of-fit test and calibration curves. The H-L test yielded *χ^2^* = 6.61, *df* = 8, *p* = 0.58 in the training set (Figure 3A), and *χ^2^* = 5.10, *df* = 8, *p* = 0.75 in the validation set (Figure 3B), indicating no significant discrepancy between the model-predicted probability and the actual incidence of ENI.

**Figure 3:**
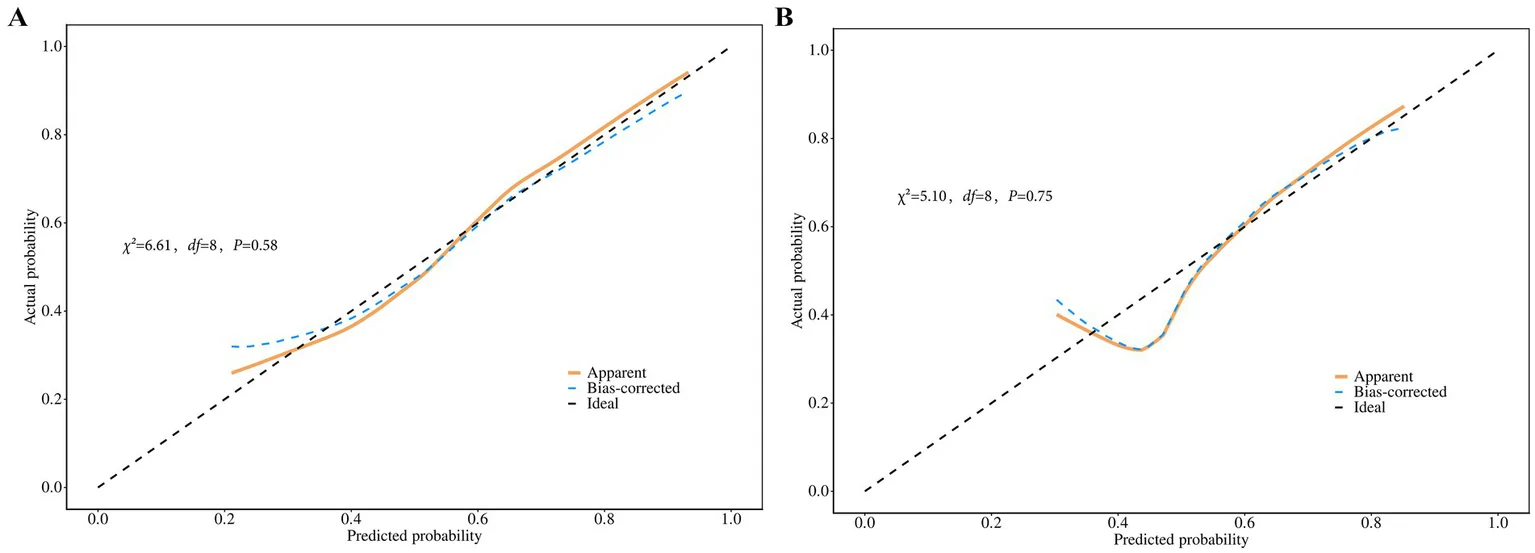
Calibration curves for the predictive model. Calibration curves were plotted to assess the agreement between predicted and actual probabilities of the outcome. The Hosmer-Lemeshow test was used to evaluate the goodness-of-fit. **(A)** Calibration curve for the training cohort. The orange line represents the apparent calibration, the blue dashed line the bias-corrected calibration, and the black dashed line the ideal reference. The Hosmer-Lemeshow test yielded *χ^2^* = 6.61, *df* = 8, *p* = 0.58, indicating good calibration. **(B)** Calibration curve for the validation cohort. The corresponding Hosmer-Lemeshow test showed, *χ^2^* = 5.10, *df* = 8, *p* = 0.75 suggesting acceptable calibration.

Plotted calibration curves indicated that both the unadjusted curve and bias-adjusted curve derived from the training cohort aligned well with the standard ideal reference line. For the validation cohort, the calibration trend roughly matched the ideal benchmark, with minor discrepancies only appearing within the range of high predicted probability. Such observational results revealed that this prediction model attained satisfactory overall calibration performance, showing high coherence between model-estimated risks and real clinical endpoints.

#### Discriminative ability of the prediction model

ROC analysis was performed to quantify discriminative ability. The AUC was 0.77 (95% CI: 0.69–0.85) for the training cohort and 0.70 (95% CI: 0.57–0.84) for the validation cohort (Figure 4). The model showed moderate discrimination and minimal overfitting, with stable performance for ENI risk stratification in sarcopenic elderly multimorbid patients.

**Figure 4:**
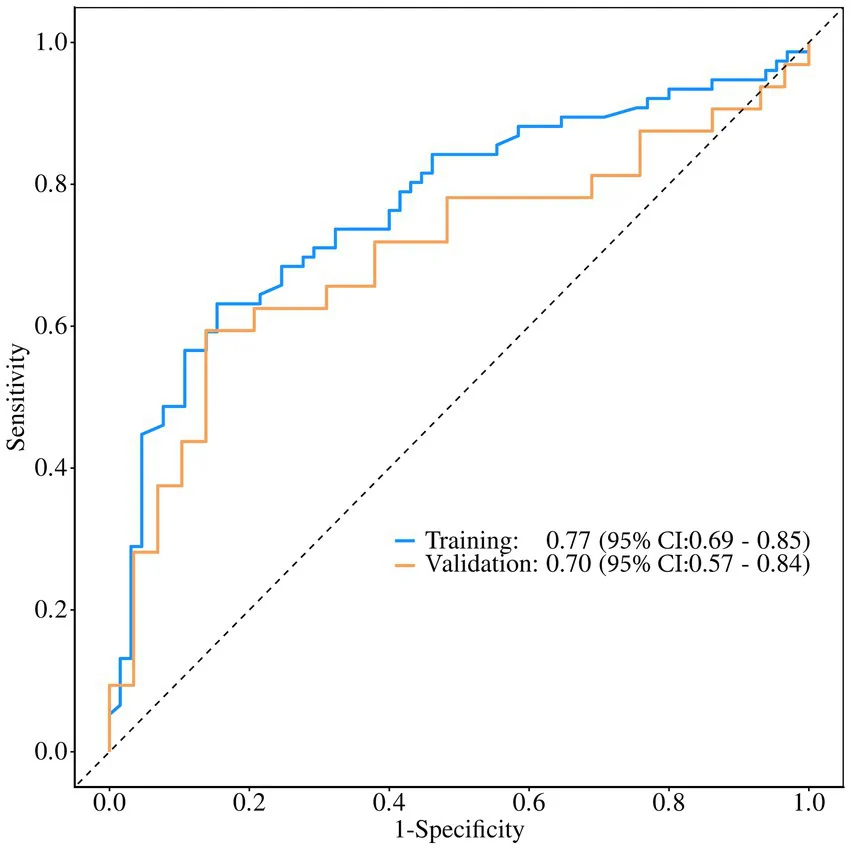
ROC curves of the prediction model in the training and validation sets. The AUC was 0.77 (95% CI: 0.69–0.85) for the training set and 0.70 (95% CI: 0.57–0.84) for the validation set. The diagonal dashed line represents the reference line for random prediction.

#### Clinical utility of the prediction model

DCA was conducted to assess the clinical utility of the prediction model. Within the range of clinically meaningful risk thresholds, the net benefit curve of the model outperformed both the “treat all” and “treat none” reference curves in the training and validation sets. These findings demonstrated that using the model to guide risk stratification and targeted intervention for ENI among elderly comorbid patients with sarcopenia could achieve higher clinical net benefit than routine empirical management strategies. The treat-all strategy yielded no positive net benefit at thresholds higher than 0.55 in the training cohort (Figure 5A) and higher than 0.52 in the validation cohort (Figure 5B), whereas the model maintained superior positive net benefit across a substantially broader threshold range covering routine clinical decision-making scenarios. This indicates that the model retains good practical performance across the most frequently used clinical intervention threshold probabilities, and can serve as a robust tool to support clinical decision-making.

**Figure 5:**
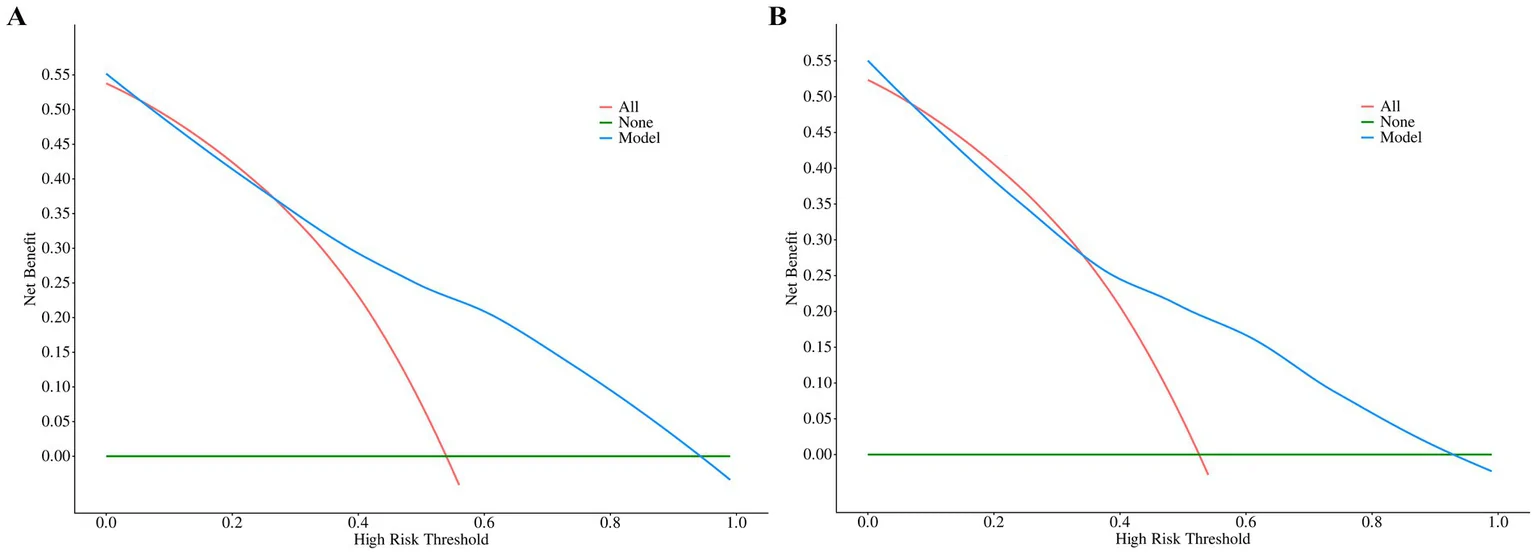
DCA curves of the model in training **(A)** and validation **(B)** sets. Blue = model, red = treat all, green = treat none. The model yields higher net benefit than reference strategies across most thresholds.

#### SHAP analysis results

SHAP analysis was applied to interpret the predictive mechanism of the established nomogram model. The SHAP value of each feature was calculated to quantify the magnitude and direction of its influence on the predictive risk probability.

##### Feature importance ranking based on the mean SHAP value

To quantify the contribution of each variable to model prediction, the SHAP approach was adopted for model interpretability analysis. The mean absolute SHAP value mean (|SHAP value|) was used to reflect the overall impact strength of each variable on ENI prediction. The results showed that the contribution of variables to model prediction ranked from high to low as follows: concomitant psychiatric disorders, RBC, ADL, CRP, and age (Figure 6A).

**Figure 6:**
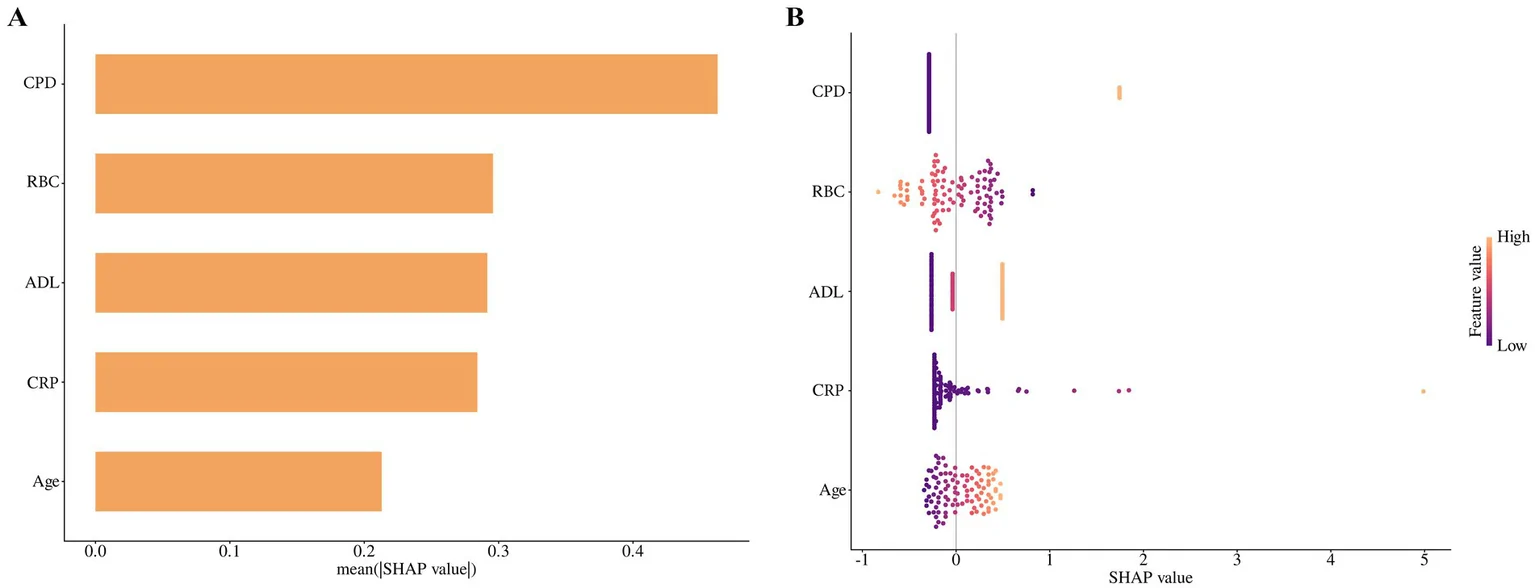
SHAP analysis of the predictive model. **(A)** Mean absolute SHAP values showing the overall importance of each feature, ranked by their contribution to model predictions. **(B)** SHAP summary plot illustrating the direction and magnitude of each feature’s impact on individual predictions. The color gradient represents feature values (low to high), with points positioned by their SHAP value to show positive or negative effects on the outcome. ADL, activities of daily living; CPD, concomitant psychiatric disorders; CRP, C-reactive protein; ENI, enteral nutrition intolerance; RBC, red blood cell count.

##### Model interpretability analysis based on SHAP Beeswarm plot

The SHAP beeswarm plot was used to further interpret the prediction model and visualize the direction and magnitude of each variable’s impact on the risk of ENI in elderly patients with comorbidities and sarcopenia (Figure 6B). Higher CPD positivity and more severe ADL impairment produced markedly positive SHAP values, serving as the top two risk-promoting factors. Elevated RBC levels were associated with negative SHAP values, exerting a protective effect against ENI. Advanced age slightly increased ENI risk, while CRP showed the weakest overall predictive contribution.

## Discussion

Elderly patients with comorbidities and sarcopenia frequently present with multiple organ function decline, malnutrition, and reduced gastrointestinal tolerance (22, 23). EN serves as an essential component of clinical management for this population, and the occurrence of ENI markedly increases the risk of adverse clinical outcomes (24–26). Existing studies have mainly focused on the screening, intervention and nutritional formula recommendation for sarcopenia (1, 2, 27). Nevertheless, most existing studies focus on the screening, intervention and nutritional regimen recommendations for sarcopenia, while few studies have developed individualized risk prediction tools for ENI in elderly patients with comorbidities and sarcopenia. Moreover, limited studies have integrated functional status, psychiatric conditions and hematological markers to establish predictive models.

Elderly patients with comorbidities and sarcopenia exhibit substantial clinical heterogeneity, and a unified nutritional management model is often insufficient to match individual tolerance and risk stratification. This highlights the urgent need to develop a targeted risk stratification tool. Based on real-world data from 202 elderly patients with comorbidities and sarcopenia, the present study identified concomitant psychiatric disorders, severe ADL dependence and decreased RBC as independent risk factors for ENI through univariate and multivariate logistic regression analyses, and further established a nomogram prediction model. The model reached an AUC of 0.77 in training data and 0.70 in validation data, showing reliable discrimination and calibration for calculating individual ENI risk. Supplementary SHAP analysis illustrated the effect size, tendency and varied influences of all predictive variables. This practical quantitative instrument assists early screening of vulnerable patients and custom feeding planning, supporting targeted nutritional care for sarcopenic elders with multiple concurrent illnesses.

This study identified concomitant psychiatric disorders as a critical risk factor for ENI in elderly patients with comorbidities and sarcopenia (OR = 7.63, *p* = 0.013). In the feature importance ranking based on mean SHAP values, psychiatric-related variables constituted the dominant influencing factor. The SHAP beeswarm plot visually showed that patients with concomitant psychiatric disorders presented markedly positive SHAP values, confirming the detrimental effect of psychiatric comorbidity on gastrointestinal function (28).

In older patients diagnosed with sarcopenia, emotional and cognitive dysfunction is closely linked to impaired gastrointestinal function and elevated susceptibility to enteral nutrition intolerance, primarily via disrupted gut-brain axis homeostasis. Clinical studies have demonstrated that patients experiencing their first episode of major depression present remarkable compositional alterations in intestinal flora, including variations in microbial alpha and beta diversity, as well as abnormal proportions of Lachnospiraceae and Bacteroidaceae taxa, which are clinically correlated with individual cognitive performance (28). Such microbial dysbiosis not only worsens cognitive deficits among depressed individuals but also compromises intestinal mucosal barrier integrity, gastrointestinal peristalsis and nutrient absorption capacity, thereby destabilizing intestinal physiological function and reducing tolerance to enteral feeding (29, 30). Sustained negative emotional states, including depression and anxiety, trigger persistent systemic stress responses that activate neuroendocrine and inflammatory pathways throughout the gut-brain axis, exacerbating intestinal mucosal inflammatory damage and further lowering the physiological threshold for enteral nutrition adaptation (31, 32). Beyond microecological and neuroinflammatory perturbations, emotional disorders increase the risk of nutrition intolerance through deteriorated physical function. Epidemiological data from middle-aged and elderly Chinese populations support that depressive symptoms aggravate sarcopenia progression by inducing disabilities in activities of daily living (33), a progressive correlation that has been further validated in national longitudinal cohort investigations (34). Concurrent cognitive impairment disrupts physiological bidirectional signal transmission along the gut-brain axis, which weakens intestinal digestive and absorptive capacity. This pathological change contributes to common enteral nutrition intolerance manifestations, such as abdominal distension and malabsorption, which conform to the unified clinical diagnostic criteria for adult enteral nutrition intolerance established by current expert consensus (17). Existing basic research also indicates that molecular mechanisms governing skeletal muscle aging and intestinal inflammatory hypoxia jointly exacerbate the interactive pathological loop covering emotional disturbance, cognitive decline, sarcopenia and gastrointestinal injury, which further increases the incidence of adverse nutritional outcomes in elderly patients (32, 35). Overall, emotional and cognitive impairments serve as crucial modifiable risk factors for enteral nutrition intolerance in elderly sarcopenic individuals. These findings suggest that routine psychological and cognitive assessments should be incorporated into daily nutritional management procedures, enabling early identification and targeted intervention to reduce enteral nutrition intolerance in high-risk elderly populations (17). In the current predictive model analysis, multivariate logistic regression analysis demonstrated a relatively wide 95% confidence interval of 1.54–37.75 for psychiatric comorbidity, which was primarily attributable to the limited sample size of the corresponding subgroup and the resultant reduced accuracy of effect size estimation. Nevertheless, internal validation based on 1,000 Bootstrap resampling procedures confirmed the favorable discriminatory and predictive performance of the established model, supporting the reliability of psychiatric and cognitive dysfunction as core predictive indicators for enteral nutrition intolerance. Considering the inherent limitations of the single-center study design and insufficient sample volume, the exact magnitude of the risk effect of psychological-cognitive impairment cannot be fully generalized. Further large-sample, multicenter prospective studies are therefore required to validate and optimize the current predictive model and strengthen its clinical applicability.

This study showed that among elderly patients with comorbidities and sarcopenia, those with severe ADL dependence had a nearly threefold higher risk of ENI (OR = 2.97, *p* = 0.018). SHAP analysis revealed severe ADL dependence carried a high mean SHAP value, and the matching beeswarm plot showed predominantly positive SHAP values among patients with severe ADL dependence, verifying its prominent positive effect on ENI in line with multivariate logistic regression findings. Beyond locomotion, skeletal muscle acts as a key endocrine organ that releases exercise-induced myokines (e.g., irisin) into the bloodstream to regulate intestinal microecology, barrier integrity and neuro-metabolic homeostasis, which are essential for normal gastrointestinal function (36). Preserved physical activity and ADL sustain skeletal muscle endocrine function. Consistent exercise enriches beneficial intestinal taxa such as Bifidobacterium, maintains intestinal barrier integrity, upregulates brain-derived neurotrophic factor (BDNF), alleviates neuroinflammation, and optimizes systemic metabolism, thereby stabilizing gastrointestinal digestive and absorptive capacity (37, 38). Conversely, elderly comorbid sarcopenia patients with impaired ADL present diminished spontaneous movement, which suppresses skeletal muscle myokine secretion. Insufficient myokine release induces intestinal dysbiosis, barrier dysfunction and systemic low-grade inflammation. Coupled with impaired neuroplasticity and dysregulated metabolism, these abnormalities disrupt gastrointestinal neural and metabolic regulation, compromise intestinal tolerance, and consequently increase the risk of enteral nutrition intolerance (39, 40). Accumulating clinical and preclinical evidence supports the close crosstalk among physical performance, skeletal muscle function, gastrointestinal homeostasis and neuro-metabolic status (41, 42). ADL impairment disrupts brain-gut-muscle axis balance in older sarcopenic patients, triggers gastrointestinal dysfunction, and serves as a pivotal risk factor for enteral nutrition intolerance. Previous studies have demonstrated that impaired ADL reflects systemic frailty and muscular dysfunction in older adults. Prolonged bedridden status and limited mobility inhibit gastrointestinal peristalsis and reduce swallowing coordination. Meanwhile, frailty-related chronic inflammation further impairs the intestinal mucosal barrier, collectively exacerbating the development of ENI (10, 33, 35). These findings are highly consistent with our results indicating that severe ADL dependence is associated with ENI in elderly patients with sarcopenia and multiple comorbidities.

This study confirmed that reduced RBC count was significantly associated with ENI and served as an independent protective factor for ENI in elderly patients with comorbidities and sarcopenia (OR = 0.56, *p* = 0.046). In the SHAP feature importance ranking, RBC count was ranked third among all predictive indicators. The SHAP beeswarm plot showed that patients with low RBC levels generally had positive SHAP values, whereas those with high RBC levels tended to have negative SHAP values, clearly illustrating the directional impact of RBC on ENI risk in elderly patients with comorbidities and sarcopenia. Under anemic conditions, insufficient microcirculatory perfusion in the gastrointestinal mucosa and abnormal mitochondrial energy metabolism weaken the intestinal mucosal repair capability. Meanwhile, hypoxia-related inflammation accelerates muscle wasting, thereby forming a vicious cycle of anemia–sarcopenia–gastrointestinal dysfunction (32, 43).

Age and CRP were significantly correlated with EN intolerance in univariate analysis, and both variables were retained after LASSO dimension reduction. However, neither was an independent predictor in multivariate regression. Both variables were still retained in the final nomogram based on AIC. Unlike conventional P value-driven variable selection, AIC-based modeling prioritizes overall model fitting and predictive performance by preserving variables with marginal predictive contributions, which minimizes information loss and improves model generalizability in clinical prediction research (44). The inconsistent predictive performance of CRP across statistical models is biologically plausible and supported by recent evidence. Elevated CRP, a reliable marker of chronic low-grade inflammation, is closely associated with the prevalence and progression of sarcopenia in older adults (45). A national cohort study of Chinese older adults further confirmed that increased CRP and multiple chronic comorbidities independently elevate sarcopenia risk (46). These findings explain the positive univariate association between CRP and EN intolerance in the present study, suggesting that sarcopenia-related chronic inflammation may impair gastrointestinal stability and increase susceptibility to EN intolerance. Importantly, CRP elevation in multimorbid older adults reflects non-specific systemic stress rather than gastrointestinal-specific inflammation. A recent study of community-dwelling octogenarians reported a weak and condition-dependent association between comorbidity burden and elevated inflammatory markers (47). In aged populations, higher CRP comprehensively results from aging-related inflammation, chronic disease accumulation, subclinical infection, frailty, anemia, and tissue hypoxia (47). After adjusting for these confounders in multivariate analysis, such non-specific inflammatory backgrounds were statistically controlled, abolishing the independent predictive value of CRP for EN intolerance. Similarly, advanced age represents progressive physiological decline and higher disease vulnerability. Although aging correlates with sarcopenia and adverse clinical outcomes, it cannot independently induce gastrointestinal dysfunction and subsequent ENI. Although age and CRP lack independent predictive value, they provide valuable auxiliary information for individualized risk stratification. Retaining these two variables according to AIC standards effectively enhances the overall predictive efficacy and robustness of the nomogram, which conforms to standardized clinical prediction model construction.

The present work carries distinct innovations in both research methodology and clinical practice. Prior relevant investigations seldom took the clinical heterogeneity of sarcopenic seniors complicated by multiple chronic illnesses into consideration, while our research specifically targets this clinically typical group whose traits are highly consistent with real clinical scenarios. We introduced SHAP interpretative analysis, combined with the sorting of average SHAP values and beeswarm diagrams for visual presentation. This approach enabled us to quantify how strongly each risk factor impacts enteral nutrition intolerance, as well as clarify the positive or negative influence of every variable. The above method successfully addresses the well-known limitation of low interpretability seen in traditional prediction models. Combining logistic regression, nomogram plotting and interpretable machine learning, we built an original risk evaluation tool with steady predictive performance and good clinical operability (16). It supplied an objective basis for nutritional intervention among elderly sarcopenic patients with multiple comorbidities, helping break the mutual deterioration loop of sarcopenia and malnutrition, and laid theoretical grounds for personalized feeding intervention plans.

This retrospective observational study was conducted at a single clinical center with all enrolled patients sourced from one institutional population, without external validation across multicenter independent cohorts. Accordingly, the extrapolation ability and general applicability of the established nomogram need to be verified in diverse external patient groups. In addition, all data in this study were extracted from routine clinical electronic medical records, which precluded the complete collection of several potential confounding factors associated with enteral nutrition intolerance and may result in residual confounding bias. Further well-designed prospective multicenter studies with standardized and exhaustive data collection procedures are therefore essential to validate the predictive performance and optimize the clinical applicability of this model.

## Conclusion

Based on clinical data from 202 elderly multimorbid patients with sarcopenia, this study identified independent risk and protective factors for ENI and constructed a nomogram-based ENI risk prediction model. The model achieved an AUC of 0.77 in the training set and 0.70 in the internal validation set, accompanied by acceptable calibration and detectable net clinical benefit, allowing for quantitative individual ENI risk stratification. Composed of routinely accessible clinical variables, this model shows moderate predictive efficacy and favorable interpretability, and may offer quantitative reference for individualized nutritional intervention planning. Further external validation in multicenter cohorts is required before widespread clinical application.

## Data Availability

The raw data supporting the conclusions of this article will be made available by the authors, without undue reservation.
