# Supplementary material for: Screening of risk factors and construction of a prediction model for enteral nutrition intolerance in elderly patients with comorbidities and sarcopenia
Source: Front Nutr. 2026 Oct 1;13:1917057. doi: 10.3389/fnut.2026.1917057 (PMC13668505; doi:10.3389/fnut.2026.1917057)
Supplement: Supplementary file 1 [file Table_1.DOCX]

**Supplementary Table 1. LASSO regression coefficients of screened predictive variables for enteral nutrition intolerance**

| Variable | LASSO coefficient (β) |
| --- | --- |
| Age  BMI  CRP  RBC  CPD  ADL | 0.021  −0.001  0.006  −0.193  0.981  0.433 |

LASSO coefficient (*β*) represents the regression coefficient of each variable after hierarchical penalized LASSO logistic regression. Variables: Age, patient age; BMI, body mass index; CRP, C-reactive protein; RBC, red blood cell count; CPD, concomitant psychiatric disorders; ADL, impaired activities of daily living.
